# Who follows through? Different factors predict initial commitment vs. Following through in a national survey of organ donor registration

**DOI:** 10.1371/journal.pone.0302587

**Published:** 2024-05-29

**Authors:** Michelle Z. Yang, Paschal Sheeran

**Affiliations:** 1 Department of Psychology and Neuroscience, University of North Carolina at Chapel Hill, Chapel Hill, NC, United States of America; 2 Department of Psychology and Neuroscience, Chapel Hill and Lineberger Comprehensive Cancer Center, University of North Carolina at Chapel Hill, Chapel Hill, NC, United States of America; Queen Mary University of London School of Business and Management, UNITED KINGDOM

## Abstract

**Objective:**

Little research has investigated factors that determine whether people falter in the face of an obstacle or successfully follow through on an initial commitment to act. We integrated multiple theories (the Reasoned Action Approach [RAA], Prototype Willingness Model, and anticipated regret theory) to test which factors predict initial commitment to register as an organ donor and to discover whether different factors predict initial commitment vs. following through with registration.

**Methods:**

Participants from a nationally representative UK sample (*N* = 1,008) reported their beliefs about organ donation and indicated their decision to register. An obstacle that participants could not foresee was that they had to complete registration in a second survey 3 days after making their initial commitment.

**Results:**

Findings showed that 14.8% of participants followed through, 19.7% demonstrated initial commitment, and 65.5% declined to register. Linear discriminant function analysis derived two functions that distinguished these registration patterns. The first function discriminated participants who declined to register from the other groups. The declined group had lower scores on RAA variables compared to their counterparts. The second function distinguished participants who made an initial commitment to register from those who followed through. Follow-through was associated with less anticipated negative affect, more favorable descriptive norms, and stronger identification with organ donors.

**Conclusions:**

The present findings indicate that even modest friction leads to a large reduction in follow-through. Moreover, different factors influence initial commitment vs. following through. Whereas RAA variables predicted initial commitment, following through was a function of anticipated negative affect and social processes.

## Introduction

Through organ donation, everyone has the power to save lives. Kidneys and livers are the organs with the highest demand, and the number of people waiting for organs far exceeds the number of transplants performed. As of 2022, 79% of the 133,319 patients on the National Transplant Waiting List are still waiting for a donor and, compared to 2021, there has been a stark 77% decline in the number of transplant recipients. According to Donate Life America, 95% of Americans are supportive of organ donation, yet only 54% were registered donors as of 2017, highlighting a discrepancy between beliefs about organ donation and registration as an organ donor. Rates of organ donor registration in 2022 marked the twelfth consecutive year of declining registration in the US [1]. Given the urgent shortage of available organs, it is crucial to identify the factors that predict organ donor registration to aid the design of interventions that could increase the supply of organs [2].

### The intention-behavior gap, initial commitment, and follow-through in organ donation

**Fig 1:**
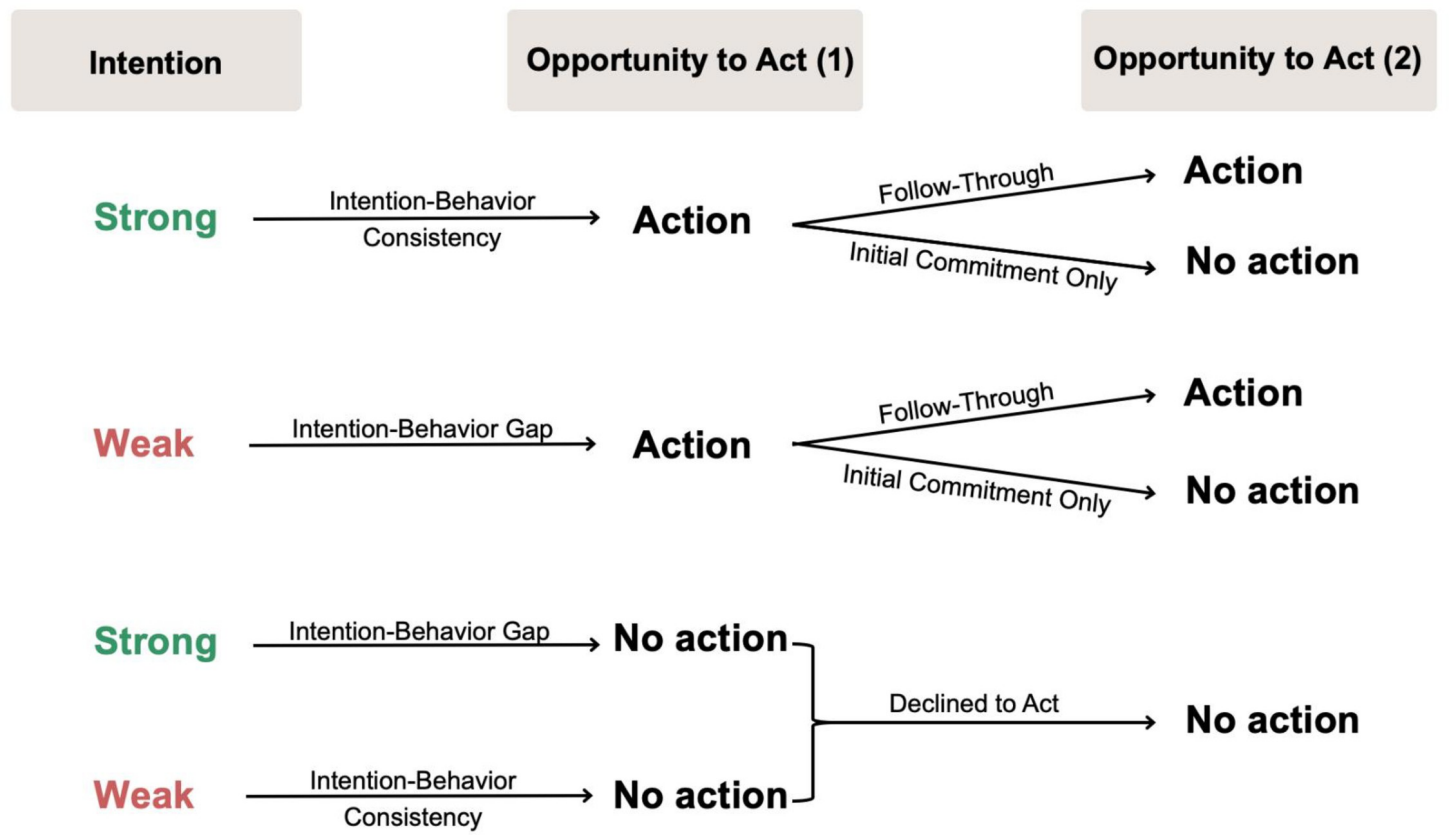
Distinguishing research on the intention-behavior ‘Gap’ from initial commitment and follow-through.

Most research on organ donor registration involves predicting willingness to become an organ donor and intentions to register [3–6, see 7, for review]. Although understanding intentions is helpful in determining whether a person will register as an organ donor, it is well-established that intentions are not always translated into behavior (intention-behavior ‘gap’) [8]. The present research is concerned with two behavioral processes–*initial commitment* and *follow-through*–that can occur in the wake of the intention-behavior gap. Fig 1 outlines how research on the intention-behavior gap, initial commitment, and follow-though are distinct. Research on the intention-behavior gap is concerned with whether people act in line with their intentions. Intention-behavior consistency occurs when people with strong intentions subsequently act and people with weak intentions do not act; the intention-behavior gap arises when people with strong intentions do not act and people with weak intentions perform the behavior. Research on initial commitment and follow-through, on the other hand, is concerned with the relationship between an initial opportunity to act and a subsequent opportunity to act.

In the present study, participants completed a survey on organ donor registration and were given two opportunities to register. As is common in organ donor registration research [9–11], we used clicking on the organ donor registration hyper-link as the index of registration behavior. The initial opportunity to act occurred at the end of the survey. Participants who acted (i.e., indicated that they wished to register) were told that it was not feasible to complete registration at this time and that they would receive an email with the link they could use to register 3 days later (i.e., the second opportunity to register). Participants who took up the initial opportunity but not the second opportunity to register can be deemed to have demonstrated initial behavioral commitment only (hereafter, *initial commitment*). For these participants, an unexpected setback in registering as an organ donor (friction) disrupts their registration efforts. Participants who take up both the initial and second opportunity to register can be said to exhibit *follow-through* on their initial commitment. Participants who did not take up the initial opportunity to register can be deemed to have *declined* registration. (The institutional IRB deemed it coercive to give participants who initially declined to register a second opportunity to register.).

So far as we are aware, research to date has not examined factors that differentiate participants who decline to act vs. demonstrate initial commitment vs. follow-through for organ donor registration decisions. The literature on behavioral commitment identifies factors that differentiate those who make an initial commitment vs. those who decline immediately (e.g., asking for people’s opinions about a charitable cause prior to donation requests) [12], and indicates that initial commitment increases the likelihood of subsequent performance (e.g., foot-in-the-door” technique) [13]. However, initial commitment is not always realized in subsequent action [see 14, for review]. The use of commitment devices (e.g., making a behavioral contract [15]) has been shown to foster persistent behavior, but the mechanisms underlying the effectiveness of such devices are unclear [16]. One proposed pathway involves leveraging normative pressure [17]. Another route is through attitudes: people may change their minds about registering as an organ donor the more they think about its negative consequences [18]. Given the lack of previous research on mechanisms underlying initial commitment vs. follow-through for organ donation, we turned to three established models of health behavior–the Reasoned Action Approach, Anticipated Regret theory, and the Prototype Willingness Model to explore whether variables specified by these theories could discriminate patterns of registration behavior (declined vs. initial commitment vs. follow-through).

#### The reasoned action approach and anticipated regret

One model that has been frequently used to study organ donor registration is the Theory of Planned Behavior (TPB); TPB variables (e.g., attitudes, subjective norms, and perceived behavioral control) are effective at predicting organ donation intentions, with attitudes and subjective norms predicting up to 88% of variance [19]. The most recent iteration of the TPB is the Reasoned Action Approach (RAA), which decomposes the components of the TPB into subcomponents: affective and cognitive attitudes, injunctive and descriptive norms, and perceived behavioral control [20]. Meta-analyses of these subcomponents demonstrated their effectiveness at predicting various health behaviors [21]. Attitudes are divided into cognitive and affective components. Cognitive attitudes refer to the instrumental consequences of acting which, in the context of organ donation, involve beliefs about the *prosocial* consequences of organ donation. Among those who report a willingness to donate their organs, the most frequently stated motivation is the prosocial benefits that organ donation has for the organ recipient [22, 23]. Affective attitudes refer to anticipated affect from engaging in a behavior, and can have a positive or negative valence [24]. Three classes of affective attitudes could be important for organ donation. The first is the “ick factor,” or the anticipated feelings of disgust evoked by organ donation. Such negative anticipated affect is associated with weaker intentions to register as an organ donor [25–27]. Second, deliberating about organ donation could serve to remind people that they are going to die and *mortality salience* may relate to donation motivation [28]. This idea is supported by evidence that mortality salience is linked to greater concern with bodily integrity [29], and decreased organ donation intentions and behavior [30, 31]. Research also shows personal fear of death is negatively associated with willingness to register [32]. Third, becoming an organ donor could be perceived as having positive affective consequences. In particular, registration could lead people to feel proud or good about themselves. According to Bandura and Cervone [33], such *self-evaluative beliefs* are a crucial determinant of motivation. A related but distinct construct to affective attitudes is *anticipated regret*, a “negative cognition-based emotion” which refers to how much regret people believe they would feel if they did not engage in a behavior [34]. The discriminant validity of attitudes and anticipated regret is well established [34, 35], and evidence indicates that anticipated regret about not donating predicts organ donor registration independently of attitudes [36, 37].

The RAA also distinguishes between two types of social norms: injunctive and descriptive. Injunctive norms refer to the belief that other people want you to perform a behavior [38]. Descriptive norms, on the other hand, refer to beliefs about the extent to which others engage in the behavior themselves [38]. A recent experimental study showed that messages highlighting descriptive norms (e.g., “Everyday thousands of people who see this page decide to register”) led to increased registration as an organ donor compared to the control condition [39]. Researchers have also investigated the role of third type of norm, *moral norms* which refer to beliefs that a behavior is morally right or wrong [40]. Moral norms predict organ donation intentions [41] and explain additional variance in intention after TPB variables are controlled [35, 42].

The measure of PBC in the RAA is traditionally separated into two components: self-efficacy (or capacity) and controllability (or autonomy). *Self-efficacy* assesses an individual’s belief in their ability to perform a behavior whereas *controllability* captures the extent to which an individual can influence an outcome [43]. However, evidence indicates that self-efficacy and controllability comprise one latent variable [33], and that self-efficacy but not controllability predict health-related intentions and behavior [21]. Accordingly, PBC for organ donation registration is typically in terms of the ease or difficulty of registering as an organ donor.

#### The Prototype Willingness Model

The Prototype Willingness Model identifies an additional source of social influence: people’s images or *prototypes* of the type of person who engages in a behavior. Prototypes have two dimensions: evaluation and similarity. *Prototype evaluation* refers to how favorable a person’s image is of the typical individual who engages in a behavior [44]. *Prototype similarity*, on the other hand, measures how similar a person’s self-image is to the type of person who engages in a behavior [44]. Most people have little experience with organ donation, and this could make them likely to rely on prototypical similarity to, or evaluation of, organ donors to inform their decision on whether to register as a donor. The predictive validity of prototypes is well established and there is evidence that prototypes influence both intentions and behavioral *willingness* (i.e., openness to engage in a behavior that is not necessarily intended) [45]. Evidence also indicates that prototypes influence intention over and above RAA variables [46] and predict behavior even after intentions have been considered [47, 48]. Furthermore, it is suggested that consistency of behavior arises when people come to see themselves as “the kind of person who performs [behavior]” [13] and through an increased sense of connection to a cause. In other words, seeing oneself as similar to a prototypical organ donor could promote following through with an initial commitment.

#### The present study

Participants in the present study were given an initial opportunity to register as an organ donor and, if they agreed, they had to undertake a second step to follow through on that initial commitment and complete their registration. This feature of the research allowed us to investigate how people respond when there is an unexpected obstacle (or *friction* [49]) on the path to organ donor registration–and to tackle two novel research questions: (1) What factors predict initial commitment to register as an organ donor? And (2) Do different factors predict initial commitment vs. following through with registration, where “following through” is defined as choosing to register at both opportunities? To address these questions, we conducted a simultaneous exploratory analysis of 11 variables specified by multiple health behavior theories (Reasoned Action Approach, Prototype Willingness Model, anticipated regret theory) among a representative UK sample.

## Methods

### Participants

We recruited a nationally representative sample of UK participants through ICM, a large British market research company, from November 4, 2015 to November 11, 2015. Participants (*N* = 1,008) were recruited as part of a larger study and were paid in accordance with company guidelines; none of the participants were registered organ donors at the time of recruitment. There were 458 men and 550 women (*M*_age_ = 52.06 years, *SD* = 14.60). The sample was White (91.2%), Asian (4.8%), Black or Afro-Caribbean (2.6%), and 1.4% other or more than one race. Eighteen-point-nine per cent of participants had incomes below £15,000 (∼ $18,250), 21.5% had incomes between £15,000 and £24,999, 18.1% had incomes between £25,000 and £34,999, 17.6% had incomes between £35,000 and £49,999, 9.4% had incomes between £50,000 and £69,999, and 4.4% had incomes > £70,000 (10.1% preferred not to say). The research was approved by the IRB at the University of North Carolina at Chapel Hill and participants provided written informed consent.

### Procedure

**Fig 2:**
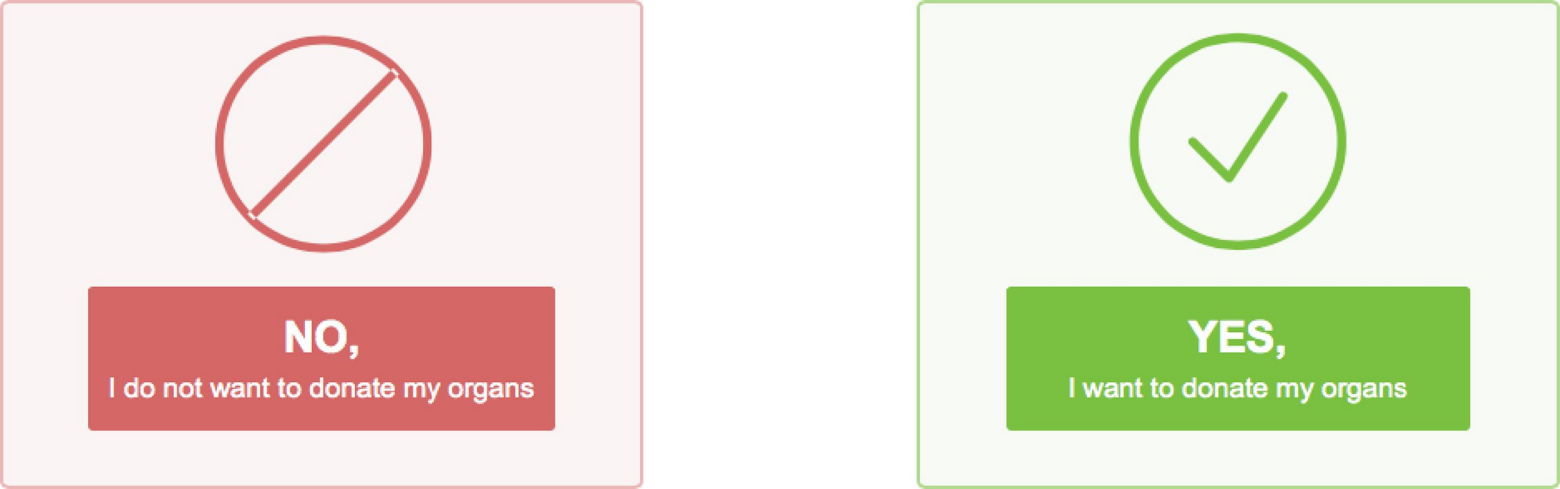
Icons used in both surveys to indicate choice to register as an organ donor.

Participants completed online surveys at two time-points over a three-day period. At Time 1, the survey measured their thoughts, feelings, and intent about registering as an organ donor. At the end of the Time 1 survey, participants were told that registering as an organ donor only takes 2 minutes and were asked to indicate if they would like to register by clicking on one of two icons indicating “Yes, I want to donate my organs” or “No, I do not want to donate my organs” (see Fig 2 for icons). Participants were told that by clicking “yes”, they would be taken to the registration website. This approach to measuring registration behavior has previously been used by health behavior researchers to obtain a proxy measure of behavior [9–11]. By operating under the pretense that clicking “yes” would initiate registration, participants’ responses allowed us to capture an initial commitment to the behavior (as participants are acting by taking a first step towards registration), rather than a self-report of intentions. However, it was not possible to direct participants who clicked “yes” to the UK organ donor registry because so doing would have meant that participants did not get paid for completing the survey (opening a new window on their computers would close the survey before participants claimed their compensation). The market research firm undertaking the research stipulated that we should wait 3 days after the Time 1 survey and email a second survey to participants who indicated, “Yes, I want to donate my organs.” The marketing company and the university’s IRB prohibited recontacting participants who indicated, “No, I do not want to donate my organs” on the grounds that doing so would be coercive. Thus, participants who clicked, “Yes, I want to donate my organs” at Time 1 were informed that they would receive a second survey. The Time 2 survey provided the identical opportunity to register as an organ donor as Time 1 but this time, participants who clicked ‘yes’ were directed to the UK organ donor registry website (https://www.organdonation.nhs.uk). Participants’ yes/no responses to the opportunity to register as an organ donor were recorded at both time-points and formed our indices of initial commitment and follow-through. It should be noted that participants had contracts with the market research company that required responses to surveys; there were no missing data, and initial commitment and follow through scores are based on participants’ actual yes/no responses. The dataset has been deposited on the Open Science Framework (*link to be inserted*).

### Measures

Participants completed measures of demographic variables before indicating their views about registering as an organ donor. Unless otherwise stated, all responses were measured on 7-point Likert scales.

#### Attitudes

In reference to the RAA, the survey contained items designed to measure attitudes. *Affective attitudes* were measured by a total of 11 items. We followed Peters’ guidance (Peters & Slovic, 2007) and used unipolar measures of discrete emotions. General affective attitudes towards organ donor registration were measured with 6 items pertaining to anticipated affect (e.g., “Registering as an organ donor would make me feel happy”; not at all—extremely) [50]. The *ick factor* was assessed with one item (e.g., “Registering as an organ donor would make me feel disgusted”; not at all–extremely”) modeled after the items O’Carroll and colleagues’ (2011) used to measure the construct [26]. Two items were used to measure *mortality salience* (e.g., “For me, registering as an organ donor would remind me that I will die one day”; strongly disagree–strongly agree), *self-evaluative beliefs*, (e.g., “Registering as an organ donor would make me feel proud of myself”; *not at all–extremely*, α = .91), and *anticipated regret* (e.g., “I would regret it if I did not register as an organ donor”; strongly disagree–strongly agree, α = .91)

*Prosocial consequences* of registration (e.g., “For me, registering as an organ donor would help to save lives”; *strongly disagree–strongly agree*), a measure of the cognitive attitude towards organ donor registration, were measured with three items.

#### Social norms

*Injunctive norm* (e.g., “Most people who are important to me think that I should register as an organ donor”; *definitely no–definitely yes*, α = .88),” *descriptive norm* (6-point scale: “Of the five people you know best, how many are organ donors ?”; α = .81), and *moral norm* (e.g., “I feel it is morally right for me to register as an organ donor”; strongly disagree–strongly agree, α = .88) were each measured with two items.

#### Perceived behavioral control

*PBC* was measured with two items pertaining to participants’ anticipated difficulty vs. ease of registering as an organ donor (e.g., “For me, registering as an organ donor would be …”; very difficult–very easy, α = .67).

#### Prototype evaluation and prototype similarity

*Prototype evaluation* (e.g., “How positive is your image of the type of person who registers as an organ donor?”; not at all positive–extremely positive)” and *prototype similarity* (e.g., “How similar are you to the type of person who registers as an organ donor?”; not at all similar–extremely similar) were both measured by two items (α = .83 and .86, respectively) [33].

#### Registration willingness, intention, and self-prediction

*Willingness* to register (e.g. “How willing are you to register as an organ donor?”; not at all willing–very willing), *intention* (e.g., “I intend to register as an organ donor”; definitely no–definitely yes), and *self-prediction* (e.g., “How likely is it that you will register as an organ donor?”; extremely unlikely–extremely likely) were each measured with two items [48].

## Results

### Exploratory factor analyses

We first tested the structure of the items measuring attitudes via factor analysis. The Kaiser-Meyer-Olkin test of sampling adequacy (.83) indicated that the data was meritorious, and Barlett’s test of sphericity (*p* < 0.001) indicated that the data was factorable. Factor analysis with oblimin rotation yielded four eigenvalues ranging from 1.18 to 5.24 (see Table 1 for factor loadings). Factor 1 included items relating to anticipated positive feelings about registering as an organ donor and was thus named *anticipated positive affect* (4 items, α = .93). Factor 2 included items relating to expectations about negative feelings accruing from registering as an organ donor, and was termed *anticipated negative affect* (5 items, α = .85). Factor 3 included items about how organ donors help others (*prosocial attitude*, 3 items, α = .90) whereas Factor 4 included items relating to how registering as an organ donor would remind participants of their own death, and was thus labeled *mortality salience* (3 items, α = .92).

**Table 1:** Factor loadings for participants’ views about registering as an organ donor.

	Components			
Item	Anticipated positive affect	Anticipated negative affect	Prosocial attitude	Mortality salience
1. Registering as an organ donor would make me feel happy	0.87			
2. Registering as an organ donor would make me feel good	0.88			
3. Registering as an organ donor would make me feel proud of myself	0.85			
4. I would feel good about myself if I registered as an organ donor	0.86			
5. Registering as an organ donor would make me feel superstitious		0.84		
6. Registering as an organ donor would make me feel jinxed		0.85		
7. Registering as an organ donor would make me feel uneasy		0.67		
8. Registering as an organ donor would make me feel uncomfortable		0.68		
9. Registering as an organ donor would make me feel disgusted		0.54		
10. For me, registering as an organ donor would help to save lives			0.94	
11. For me, registering as an organ donor would benefit others			0.95	
12. For me, registering as an organ donor would show my concern for others			0.54	
13. For me, registering as an organ donor would remind me that I will die one day				0.87
14. For me, registering as an organ donor would make me think about my own death				0.98

Note. *N =* 1008. Factor loadings < 0.3 are not displayed

Second, we ran a factor analysis to determine whether willingness, intention, and self-prediction items loaded on separate factors. The Kaiser-Meyer-Olkin test yielded an overall MSA of 0.89 and Barlett’s test of sphericity (*p* < 0.001) indicated that data was factorable. However, factor analysis resulted in only one eigenvalue greater than 1.0 indicating that all of the items involved a single construct, which we termed *intention* (α = .97).

Next, we computed correlations between predictors and outcomes (see Table 2). There was a very high correlation between moral norm and intention (*r* = .83) so we ran a factor analysis to determine whether intention and moral norms were distinct constructs. Kaiser-Meyer-Olkin analysis yielded an overall MSA of 0.93 and Barlett’s test of sphericity (*p* < 0.001) indicated that the data was factorable, but factor analysis returned only one eigenvalue greater than 1.0, suggesting that they load onto one factor. Since factor analysis revealed that moral norms could not be distinguished from intentions in our data, we excluded moral norms from our analyses as it would be problematic to predict intentions from moral norms in light of their high correlation and lack of discriminant validity.

**Table 2:** Descriptives and correlations for study variables.

Predictors	1	2	3	4	5	6	7	8	9	10	11	12	13	14
1. Anticipated positive affect														
2. Anticipated negative affect	-0.28***													
3. Prosocial attitude	0.14***	0.30***												
4. Mortality salience	0.59***	-0.21***	0.22***											
5. Injunctive norm	0.62***	-0.15***	0.12***	0.45***										
6. Descriptive norm	0.22***	-0.09**	-0.04	0.13***	0.34***									
7. Moral norm	0.81***	-0.29***	0.11***	0.57***	0.67***	0.26***								
8. PBC	0.43***	-0.23***	0.00	0.43***	0.45***	0.19***	0.47***							
9. Prototype similarity	0.63***	-0.23***	0.18***	0.57***	0.4***	0.18***	0.62***	0.38***						
10. Prototype evaluation	0.68***	-0.24***	0.07*	0.47***	0.57***	0.27***	0.71***	0.46***	0.58***					
11. Intention	0.76***	-0.32***	0.06*	0.54***	0.73***	0.26***	0.83***	0.53***	0.51***	0.67***				
12. Anticipated regret	0.72***	-0.13***	0.17***	0.45***	0.68***	0.24***	0.72***	0.38***	0.44***	0.61***	0.77***			
13. Initial commitment	0.47***	-0.32***	-0.03	0.34***	0.48***	0.22***	0.54***	0.39***	0.30***	0.41***	0.69***	0.52***		
14. Follow through	0.32***	-0.26***	-0.04	0.22***	0.34***	0.21***	0.38***	0.25***	0.24***	0.29***	0.47***	0.33***	0.57***	
Mean	4.50	2.68	4.72	5.56	3.53	1.02	4.40	4.66	5.31	4.15	3.92	3.33	0.35	0.15
*SD*	1.58	1.42	1.86	1.43	1.56	0.75	1.69	1.54	1.34	1.49	1.76	1.72	0.48	0.36

Note.

* *p* < .05.

** *p* < .01.

*** *p* < .001

### Understanding initial commitment vs. Following through with organ donor registration

Three groups could be identified based on participants’ responses at the first and second registration opportunities. The first and largest group were participants who did not click the registration link in the Time 1 survey and thus declined to register as organ donors (*Declined*, 65.5%). The second largest group clicked on the registration at Time 1 but did not register at the second registration opportunity (*Initial Commitment*, 19.7%). The third group comprised participants who clicked the link at both the Time 1 and Time 2 registration opportunities (*Follow-Through*, 14.8%). To predict group membership, predictors were entered in a direct discriminant function analysis. Two discriminant functions were computed with a combined *X*^*2*^(11, *N* = 1008) = 292.86, *p* < .001; 72.1% of grouped cases were correctly classified. After adjustment for group size, this classification is 27.1% greater than would be expected by chance.

**Fig 3:**
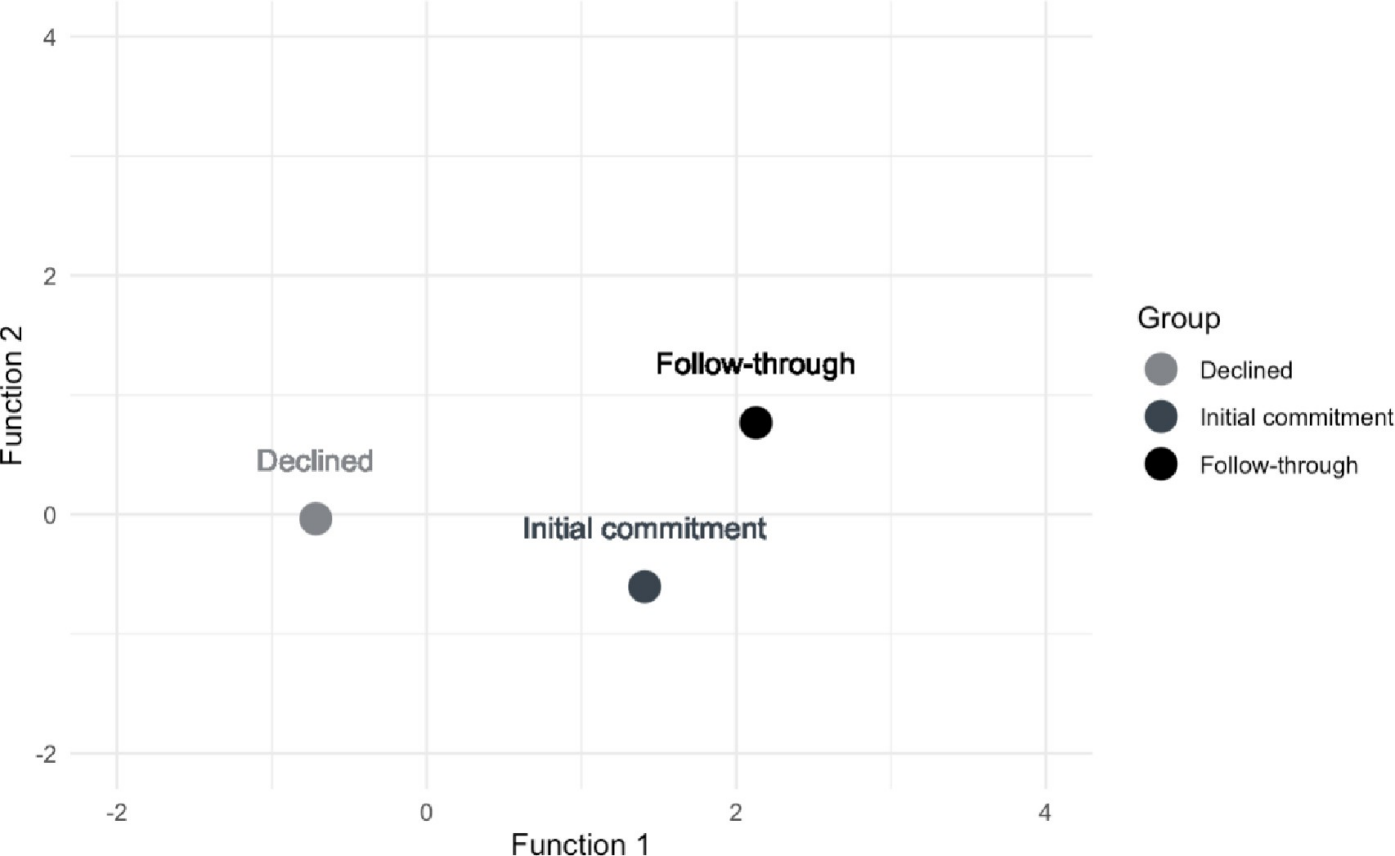
Plots of three group centroids on two discriminant functions derived from 11 predictor variables.

Fig 3 plots the group centroids on the two significant discriminant functions derived from our 11 predictor variables. The first derived function (horizontal axis) maximally distinguished the *Declined* group (centroid = -0.74) from the combination of the *Initial Commitment* and *Follow-Through* groups (centroids = 1.19 and 1.67, respectively), which we called the *Actor* group. The second function (vertical axis) distinguished the *Initial Commitment* group (centroid = 0.20) from the *Follow-Through* group (centroid = -0.21). Seven variables were significantly correlated with the first discriminant function (anticipated positive affect, prosocial attitude, injunctive norm, PBC, prototype evaluation, anticipated regret, and intention; see Table 3). We used ANOVAs to compare means for the *Declined* group vs the *Actor group* to aid interpretation of the function. As Table 2 shows, the *Declined* group had lower scores for RAA variables and prototype evaluation. That is, compared to participants who showed initial agreement to register as an organ donor, participants who declined to register had lower intentions and anticipated less positive affect about registering as a donor; they also expected less regret about not registering and felt less social pressure to register, saw fewer prosocial consequences of registration, had a less favorable image of people who register, and believed that registration was more difficult.

**Table 3:** Correlations with discriminant functions, descriptive statistics, and F-ratios for participant groups.

Predictor	Function 1	Function 2	Declined	Actor	*F*	Initial Commitment	Follow-Through	*F*
			*M (SD)*	*M (SD)*		*M (SD)*	*M (SD)*	
Intention	0.94†	0.11	-0.50 (0.79)	0.95 (0.59)	911.11***			
Anticipated regret	0.60†	0.30	-0.38 (0.87)	0.72 (0.83)	378.60***			
Injunctive norms	0.55†	-0.09	-0.35 (0.89)	0.66 (0.86)	304.52***			
Anticipated positive affect	0.53†	0.08	-0.34 (0.96)	0.65 (0.70)	293.02***			
Prototype evaluation	0.44†	-0.13	-0.30 (0.96)	0.56 (0.81)	199.12***			
PBC	0.41†	0.20	-0.28 (0.99)	0.53 (0.77)	175.10***			
Prosocial attitude	0.35†	0.10	0.24 (1.08)	0.46 (0.61)	128.62***			
Prototype similarity	0.32	-0.34†				0.30 (0.78)	0.58 (0.67)	12.31***
Anticipated negative affect	-0.34	0.43†				-0.30 (0.89)	-0.62 (0.85)	11.34***
Descriptive norms	0.24	-0.57†				0.16 (1.08)	0.50 (1.25)	7.24**
Mortality salience	-0.03	0.19†				0.00 (0.95)	-0.09 (0.92)	0.84

*Note*. † indicates significant correlation with respective function. Scores on predictor variables were standardized. PBC = perceived behavioral control.

* *p* < .05

** *p* < .01

*** *p* < .001.

Four variables were significantly correlated with the second discriminant function (anticipated negative affect, descriptive norm, prototype similarity, and mortality salience; see Table 3). ANOVAs again were undertaken to determine which variables distinguish the *Initial Commitment* and *Follow-Through* groups. Whereas mortality salience scores did not differ for the two groups, differences for the other variables were significant. Participants who followed through reported less anticipated negative affect about registration, knew more people who were registered organ donors, and showed stronger identification with organ donors compared to participants in the *Initial Commitment* group.

## Discussion

Relatively little theoretical and empirical work in health psychology and behavioral medicine has examined how people respond to obstacles to goal attainment, or tested factors that could distinguish between people who persist with, and those who abandon, striving for a goal. In the present study, we exploited a feature of the data collection process–the fact that participants could not leave the survey to visit the organ donor website–to examine how an obstacle affects striving for the goal of organ donor registration. The obstacle was not especially onerous; participants merely had to complete a second survey containing a single question 3 days after initially committing to register as an organ donor, and thereafter spend 2 minutes finalizing their registration. However, only 42% of participants who made the initial commitment followed through. Despite the considerable social and public health importance of organ donor registration, a small amount of friction led a lot of people to give up.

We tested 11 variables from multiple behavioral theories to identify predictors of both initial commitment and follow-through. Findings showed that participants were more likely to make an initial commit to registration when they reported stronger intentions to register, more positive prosocial attitudes and anticipated affect, increased social pressure, a favorable image of organ donors, higher perceived control, and greater anticipated regret about not registering. These findings support the RAA as a model of initial commitment to organ donor registration. At the same time, it is notable that factors not specified by the RAA such as anticipated regret and prototype evaluation also correlated with the discriminant function alongside intentions and PBC.

Whereas RAA variables discriminated participants in the *Initial Commitment* and *Declined* groups, a different set of factors–negative anticipated affect, descriptive norms, and prototype similarity–distinguished participants in the *Initial Commitment* and *Follow-Through* groups. Compared to those who merely made an initial commitment, participants who followed through were less likely to anticipate negative emotions about registration (i.e., feeling jinxed, uneasy, uncomfortable, or disgusted); they were more likely to have organ donors in their social circle and felt a stronger sense of identification with organ donors. These findings indicate that intentions and other RAA variables predict initial commitment but these variables do not explain whether that initial commitment culminates in follow-through. Instead, following through with donor registration behavior is explained by anticipated affect and social influences.

The conceptual and practical significance of these findings are fourfold. First, although there has been extensive theoretical treatment of how obstacles can undermine goal attainment, for example, in analyses of *friction* [49] and *sludge* [51], relatively few studies have documented the impact of obstacles on health behavior performance. An important exception is Mazar et al.’s test of the impact of travel distance to vaccination sites on rates of COVID-19 vaccination where a medium-sized, negative correlation was observed (*r* = -.36) [52]. The present study contributes to this database and shows that an ostensibly modest amount of friction thwarted registration behavior for almost 60% of participants who had made an initial commitment to register. More important, our research demonstrates for the first time, that encountering an obstacle switches the mode of action control from intentional, reasoned mechanisms of action to regulation by affective attitudes and social processes.

Second, these findings have important implications for the RAA as they would seem to demonstrate that while initial striving for a goal is under intentional control, subsequent striving that may be needed when friction is experienced, is not. It is well established that intention-behavior consistency declines as the time interval between the measurement of intentions and behavior increases (see [53, 54], for meta-analyses). However, the timescales involved in these analyses are weeks and months whereas the delay for following through in the present research was only 3 days; this suggests that time interval does not explain the poor performance of RAA variables in explaining follow-through here. Other meta-analytic evidence that may be consistent with the present findings is that RAA variables better predict infrequent actions than behaviors that are frequently performed [55]. People encounter more obstacles when behaviors are performed more often, and the present findings suggest that dealing with obstacles could undermine intentional control. Additional research is needed to corroborate our finding that friction reduces the predictive validity of intentions, and to interrogate when and why friction has this effect.

Third, none of theoretical perspectives tested in the present research (the RAA, Prototype Willingness Model, anticipated regret theory) predicts that negative anticipated affect, descriptive norms, and prototype similarity would influence following through with donor registration, as we observed. Why did anticipated negative affect and social processes bypass reasoned processes to influence persistent goal pursuit? Negative affect is known to impede goal pursuit by reducing both the accessibility and desirability of goals [56] and it is possible that anticipating negative affect about organ donor registration could thus have tarnished registration goals during the interim between initial commitment and follow-through. Recent research on social proof may explain why descriptive norms directly influenced registration behavior–normative behaviors are more likely to come to mind and are perceived as more feasible than non-normative behaviors [57]. This analysis suggests that when organ donor registration behavior is more common in people’s social circles, mental representations of the behavior are more accessible, and so individuals are more likely to follow through with registration. Finally, the influence of prototype similarity on donor registration echoes previous research showing that prototype similarity directly predicts behavior. Rivis and colleagues observed that augmenting TPB models with prototype similarity increased the variance explained in 14 health behaviors by 6% above and beyond intention [47]. Thus, social identification appears to constitute an important pathway to organ donor registration that is independent of participants’ intentions to act.

Fourth, our results also have implications for donor registration interventions. The present findings underscore the value of reducing friction in promoting organ donor registration [8], as minor obstacles can undermine registration even among committed individuals. To promote initial commitment to register as organ donors, interventions could target RAA variables such as perceived behavioral control and attitudes about organ donor registration (see [20], for a review). However, to sustain commitment over time and in the face of obstacles, interventions may need to target anticipated negative emotions about registration, descriptive norms, and prototype similarity. Improved emotion control predicts persistence with behaviors such as completing treatment for substance abuse [58] and affect regulation interventions have been found effective in promoting health behavior change [59]. For instance, planning to reappraise negative affect about attending psychotherapy (“[I’ll] tell myself this [feeling] is perfectly understandable”) increased rates of subsequent attendance for treatment [60, 61]. A similar approach could be applied to organ donor registration to reduce the impact of anticipated negative affect on following through. Research has also demonstrated that increasing feelings of hopefulness can help overcome fear of death from organ donation, and promote willingness to register as a donor [32]. Interventions could target descriptive norms either by highlighting the prevalence of organ donor registration within reference groups [62] or increasing the visibility of organ donor registration behavior—when a behavior is easily observed, individuals are more likely to construe that behavior as normative [63, 64]. Interventions to increase prototype similarity could invite participants to identify with organ donors. One approach could be to ask individuals to list the ways that they are similar to organ donors [65], or to identify what they have in common with specific individuals in their in-group who are organ donors [66].

Strengths and weaknesses of the research should be considered. Strengths include the recruitment of a large, representative sample; offering an integrative test by including variables specified by different health behavior theories; measuring attitudes towards organ donor registration specifically rather than organ donation generally, thus increasing correspondence between measures of attitude and behavior [11]; and using a task measure that had mundane realism to measure donor registration behavior (and not merely hypothetical choices). A weakness is the fact that we could not, for legal reasons, confirm that participants indeed registered as organ donors when they were directed to the NHS website. Clicking on a hyper-link is widely used to measure organ donor registration behavior [9, 10] and there is evidence that this measure may be valid. Siegal et al [11] conducted two studies, one in which participants were given paper organ donor registration forms and a second conducted online where participants clicked on a hyper-link. Findings showed no difference in registration rates between the two methods (13% vs. 10%). Nonetheless, it would be valuable for future studies to confirm subsequent registration by asking participants to show their donor cards. It is also the case that this was an exploratory, observational study. Although we recruited non-donors and offered opportunities to register after participants had completed survey items, causal relations cannot be inferred. Future studies should manipulate predictors to experimentally corroborate the findings obtained here.

There are several additional directions for future studies. First, our study examined follow-through in the face of an obstacle that lasted a relatively short period of time (i.e., 3 days). Subsequent research could investigate whether anticipated negative affect and social influence consistently discriminates initial commitment and follow-through over a longer period of time. Third, studies could control for individual differences in preference for consistency (PFC) [67], to gauge how a person’s desire to be consistent with their initial response or behavior influences follow-through. Finally, organ donor registration behavior involves unique considerations (e.g., bodily integrity, emotional impact for family members, religious beliefs) [23], which suggests that additional research is needed to determine whether affective attitudinal and social processes predict follow-through for other health behaviors (e.g., exercise regimens, attending therapy).

## Conclusions

The present research combined behavioral theories from health psychology with the behavioral economic concept of friction to analyze organ donor registration. We found that friction gravely undermined rates of follow-through with registration even among participants who had made an initial commitment to register. Tests of constructs from key behavioral theories indicated that different factors predicted initial commitment vs. following through. Whereas initial commitment was explained by RAA variables, people who follow through were characterized by less anticipated negative affect and salutary social influences. These findings have important implications for understanding behavioral persistence, for the generality of reasoned action processes, and for tailoring interventions on obstacles that participants are liable to encounter [68]. Research to corroborate the findings obtained here and test interventions that could close the gap between initial commitment and follow-through is warranted.
